# Supplementary figures and images for: Characterization of blaNDM-5-and blaCTX-M-199-Producing ST167 Escherichia coli Isolated from Shared Bikes
Source: Antibiotics (Basel). 2022 Jul 30;11(8):1030. doi: 10.3390/antibiotics11081030 (PMC9404906; doi:10.3390/antibiotics11081030)

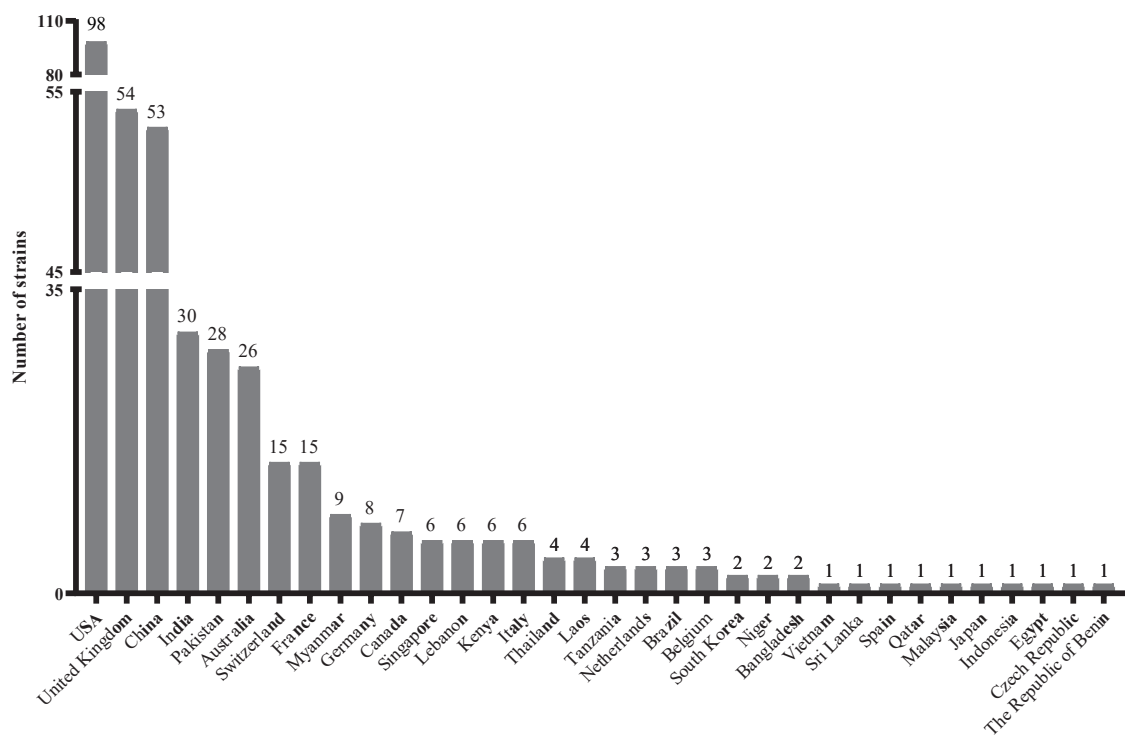

Figure S1. Number of ST 167 *E. coli* from different countries or districts

Supplement: Supplementary file 1 [file antibiotics-11-01030-s001.zip › Figure S1-2022-07-03.pdf]
